# Health economic evaluations comparing faecal microbiota transplantation with antibiotics for treatment of recurrent *Clostridioides difficile* infection: a systematic review

**DOI:** 10.1186/s13561-021-00301-7

**Published:** 2021-01-13

**Authors:** Lianna Hede Hammeken, Simon Mark Dahl Baunwall, Christian Lodberg Hvas, Lars Holger Ehlers

**Affiliations:** 1 Danish Center for Healthcare Improvements, Department of Clinical Medicine, Aalborg University, Fredrik Bajers Vej 5, DK-9220 Aalborg Ø, Denmark; 2 Department of Hepatology and Gastroenterology, Aarhus University Hospital, Palle Juul-Jensens Boulevard 99, DK-8200 Aarhus, Denmark

**Keywords:** Systematic review, Economic evaluation, Fecal microbiota transplantation, Faecal microbiota transplantation, Anti-bacterial agents, Decision making, Clinical decision-making

## Abstract

**Background:**

Faecal microbiota transplantation (FMT) is increasingly being used in the treatment of recurrent *Clostridioides difficile* infection (rCDI). Health economic evaluations may support decision-making regarding the implementation of FMT in clinical practice. Previous reviews have highlighted several methodological concerns in published health economic evaluations examining FMT. However, the impact of these concerns on the conclusions of the studies remains unclear.

**Aims:**

To present an overview and assess the methodological quality of health economic evaluations that compare FMT with antibiotics for treatment of rCDI. Furthermore, we aimed to evaluate the degree to which any methodological concerns would affect conclusions about the cost-effectiveness of FMT.

**Methods:**

We conducted a systematic literature review based on a search in seven medical databases up to 16 July 2020. We included research articles reporting on full health economic evaluations comparing FMT with antibiotic treatment for rCDI. General study characteristics and input estimates for costs, effectiveness and utilities were extracted from the articles. The quality of the studies was assessed by two authors using the Drummonds ten-point checklist.

**Results:**

We identified seven cost-utility analyses. All studies applied decision-analytic modelling and compared various FMT delivery methods with vancomycin, fidaxomicin, metronidazole or a combination of vancomycin and bezlotoxumab. The time horizons used in the analyses varied from 78 days to lifelong, and the perspectives differed between a societal, a healthcare system or a third-party payer perspective. The applied willingness-to-pay threshold ranged from 20,000 to 68,000 Great Britain pound sterling (GBP) per quality-adjusted life-year (QALY). FMT was considered the most cost-effective alternative in all studies. In five of the health economic evaluations, FMT was both more effective and cost saving than antibiotic treatment alternatives. The quality of the articles varied, and we identified several methodological concerns.

**Conclusions:**

Economic evaluations consistently reported that FMT is a cost-effective and potentially cost-saving treatment for rCDI. Based on a comparison with recent evidence within the area, the multiple methodological concerns seem not to change this conclusion. Therefore, implementing FMT for rCDI in clinical practice should be strongly considered.

**Supplementary Information:**

The online version contains supplementary material available at 10.1186/s13561-021-00301-7.

## Background

*Clostridioides difficile* infection (CDI) is an enteric infection causing symptoms ranging from diarrhoea and abdominal discomfort to toxic megacolon and death [1, 2]. In Europe, the incidence rate for healthcare-associated CDI has been estimated to 23 per 10,000 admissions [3]. The standard treatment for CDI is antibiotics, but 20–30% fail to achieve a sustained response and suffer recurrence [4, 5].

Current clinical guidelines recommend oral vancomycin or oral fidaxomicin for the first recurrence of CDI (rCDI) and pulse/taper oral vancomycin, fidaxomicin or faecal microbiota transplantation (FMT) for any subsequent recurrent episodes [6, 7]. FMT is a novel procedure by which minimally processed faeces from a thoroughly screened healthy donor is transferred to a patient to correct a disrupted intestinal microbiota [8]. During the past decade, FMT has gained widespread recognition as a highly effective treatment for rCDI; effect rates exceeding 90% for FMT have been reported in observational studies [9, 10], and randomised controlled trials (RCTs) have found FMT to be comparable [11] or superior to antibiotic treatments for patients with rCDI [12–14].

Healthcare systems worldwide are currently facing increasing budgetary pressures due to an ageing population and expensive medical treatments [15], and the need is rising to include both clinical and economic considerations in the decision-making when implementing new technologies [16]. Health economic evaluations may help qualify these decisions by providing an analysis of the costs and consequences of two or more alternative technologies [16]. Given the growing interest in the use of FMT for rCDI treatment [17], it becomes important to support healthcare decision-making regarding the implementation of FMT in clinical practice. Previous systematic reviews have evaluated the cost-effectiveness of providing FMT for both index CDI and rCDI and have highlighted several concerns about the methodological quality of existing health economic evaluations [18, 19]. However, the impact of these concerns on the conclusions of the studies has yet to be evaluated.

The present study aimed to present an overview of and to assess the quality of health economic evaluations that compare FMT with antibiotics in the treatment of rCDI based on a systematic literature review. We further aimed to evaluate how any methodological concerns would affect the conclusions about cost-effectiveness based on a comparison with recent evidence within the area, and thereby hope to provide clear guidance for healthcare decision-makers.

## Materials and methods

This systematic review was conducted in accordance with the PRISMA guidelines for reporting of systematic reviews and meta-analyses [20].

### Eligibility criteria

Predefined criteria were applied to identify relevant studies. We included full health economic evaluations comparing FMT with any antibiotic treatment strategy for rCDI. A health economic evaluation was defined as a study comparing both the costs and consequences of alternative treatments, i.e. cost-benefit, cost-effectiveness or cost-utility analyses [16]. We excluded studies including only costs or consequences and article types other than research articles, e.g. reviews, editorials and conference abstracts.

### Search strategy

A systematic literature search was conducted up to 16 July 2020 for all available literature in the following databases: PubMed, Embase, the Cochrane Library, Cinahl, Scopus, EconLit and the NHS Economic Evaluation Database. If the database allowed it, the search was limited to English and Danish language. No other restrictions were made. A combination of the following search terms was applied: ‘*Clostridium difficile* infection’ AND ‘fecal microbiota transplantation’ AND (‘economic evaluation’ OR ‘cost-benefit’ OR ‘cost-effectiveness’ OR ‘cost-utility’). The search terms were adjusted to the specific databases and consisted of a combination of thesaurus terms and free-text search. The authors have previously reported a preliminary study using a similar search strategy in a conference abstract [21]. The complete search strategy used in the present systematic review is available in Additional file 1.

### Study selection

After searching the specified databases, duplicates were removed using the reference managing software RefWorks (ProQuest, Michigan, USA). Remaining duplicates were identified by comparing the title, author, year and journal of the studies and were manually removed. After the exclusion of duplicates, records were screened for inclusion based on the title and abstract. The full text of the remaining studies was then assessed. Two authors (LHH and SMDB) independently conducted the screening and assessment for inclusion of records, and any disagreements were resolved by dialogue between the two authors.

### Data extraction

The following data on study characteristics were extracted from the included records: author and year of publication, setting, analytic method, time horizon, perspective, outcome measure, patient population, intervention, control treatment, willingness-to-pay threshold, results and the conclusion regarding cost-effectiveness. Also, input estimates applied for utilities, effectiveness and costs were extracted to a separate table. Costs were both presented as original values and converted to 2019-level Great Britain pound sterling (GBP). Extrapolation was done by applying a country-specific consumer price index [22]. Afterwards, purchasing power parity adjusted exchange rates were used to convert the estimates into GBP [23].

### Quality assessment

Methodological quality was assessed using the Drummond ten-point checklist for assessing economic evaluations [16]. The checklist consists of ten essential items and several subitems related to, e.g., the research aim and choice of comparators, the effectiveness measure, costs and outcome measures as well as the results and uncertainties of the analysis [16]. Two authors (LHH and LHE) assessed each of the studies and agreed on a final assessment. The results from the quality assessment were reported for each checklist item and categorised into the following four answers: yes (adequate), no (not adequate), cannot tell (unclear) or not applicable based on compliance with the checklist.

## Results

### Study selection

**Fig. 1:**
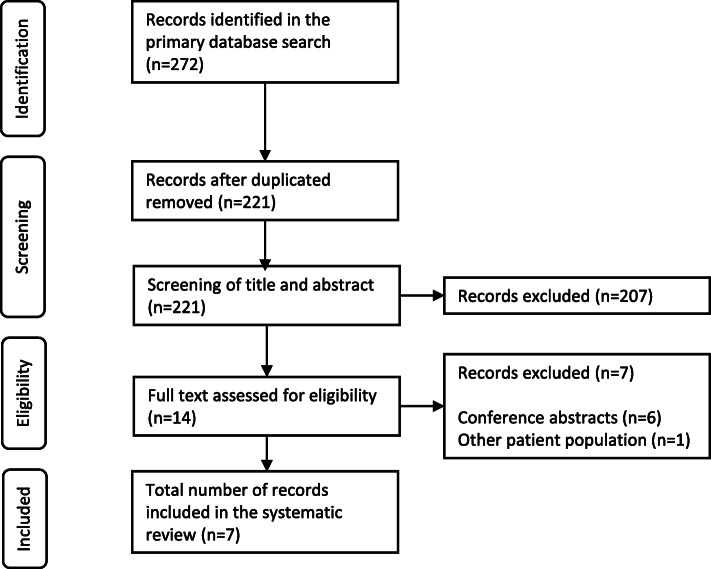
Study selection flow-chart. The chart is adapted from the PRISMA-guidelines for reporting of systematic reviews [20]

A total of 272 records were identified in the systematic literature search (Fig. 1). Fifty-one duplicates were removed, and the remaining 221 records were screened based on the eligibility criteria. Among these, 207 were excluded as they were not full health economic evaluations, did not compare FMT with antibiotic treatment or did not focus on rCDI. Thus, the full text of a total of 14 records was screened, and seven of these were included in the systematic review [24–30].

### Study characteristics

A schematic presentation of the general study characteristics is provided in Additional file 2. The studies originated from the United States (*n* = 3) [24, 25, 29], Canada (*n* = 1) [26], Australia (n = 1) [27], France (n = 1) [28] and the United Kingdom (n = 1) [30] and were published between 2014 and 2020. All studies were cost-utility analyses based on decision-analytic modelling using either decision trees (*n* = 4) [24, 25, 28, 29] or Markov models (*n* = 3) [26, 27, 30]. The number of previous rCDI episodes differed between studies, ranging from the first to the third rCDI at inclusion.

All studies included FMT administered by colonoscopy, and some also included nasoduodenal/−gastric infusion (*n* = 5) [24, 26–28, 30], enema (n = 3) [24, 26, 28] or oral capsules (*n* = 1) [29]. The treatment of additional recurrences was either reported as repeat FMT or a shift to oral vancomycin. In all studies, the control treatment was oral vancomycin in different dosages and treatment regimens. Fidaxomicin was applied in most studies (n = 5) [24, 26, 28–30], whereas two studies included oral metronidazole [24, 26], and one study included the combination of oral vancomycin with bezlotoxumab [29]. In case of additional recurrences, the subsequent antibiotic treatment strategies differed in accordance with the number of previous rCDI episodes.

The studies either applied a relatively short time horizon of 78 days to 1 year (*n* = 5) [24, 25, 28–30] or a lifelong time horizon (*n* = 1) [26], whereas the reported perspectives differed between a societal (*n* = 2) [24, 28], a third-party payer (n = 2) [25, 29] and a healthcare system perspective (n = 2) [26, 30]. The willingness-to-pay threshold ranged from 20,000 to 68,000 GBP per quality-adjusted life-year (QALY).

FMT was estimated as the most cost-effective alternative in all studies. In five of the health economic evaluations, FMT strongly dominated, i.e. was both cost-saving and more effective than all the antibiotic treatment alternatives [25–27, 29, 30]. However, the results differed with respect to FMT delivery method; FMT by colonoscopy was cost-effective in four of the studies [24–26, 29], whereas FMT by nasoduodenal/−gastric infusion was cost-effective in two studies [27, 30], and FMT by enema was the most cost-effective alternative in one study [28]. If the most cost-effective delivery method was unavailable, in five of the six studies including more than one delivery method of FMT, alternative delivery methods were also considered cost-effective compared with the antibiotic treatments [26–30]. Additional file 3 presents an overview of the cost-effectiveness of the different delivery methods of FMT.

### Methodological quality assessment

The methodological quality of each study was evaluated according to the Drummond ten-point checklist for assessing economic evaluations [16]. The overall evaluation is presented in Table 1. Detailed assessments of each article are available in Additional file 4, and a table containing the inputs applied in the analyses is provided in Additional file 5.

**Table 1:** Results of the quality assessment of health economic evaluations

Item	Konijeti et al., 2014 [24]	Varier et al., 2015 [25]	Lapointe-Shaw et al., 2016 [26]	Merlo et al., 2016 [27]	Baro et al., 2017 [28]	Luo et al., 2020 [29]	Abdali et al., 2020 [30]
**Question 1. Research aim**	Yes	Yes	Yes	No	Yes	Yes	Yes
**Question 2. Alternatives**	Yes	Yes	Yes	Yes	Yes	Yes	Yes
**Question 3. Effectiveness**	Cannot tell	Cannot tell	Cannot tell	Cannot tell	Cannot tell	Cannot tell	Cannot tell
**Question 4. Identification of costs and consequences**	No	No	Yes	Cannot tell	No	No	No
**Question 5. Measurement of costs and consequences**	Cannot tell	Cannot tell	Yes	Cannot tell	Yes	Cannot tell	Yes
**Question 6. Valuation of costs and consequences**	Cannot tell	Cannot tell	Yes	Cannot tell	Yes	Cannot tell	Yes
**Question 7. Extrapolation and discounting**	NA	NA	Yes	Yes	NA	NA	NA
**Question 8. Results**	Yes	Yes	Yes	Yes	Yes	No	Yes
**Question 9. Sensitivity analyses**	Yes	Yes	Yes	No	Yes	Yes	Yes
**Question 10. Discussion**	Cannot tell	Cannot tell	Yes	No	Cannot tell	Cannot tell	Yes

The items reported in the table are based on the main topic of each item in the checklist for assessing economic evaluations reported by Drummond et al., 2015 [16]. The answers reported in the table indicate the compliance with the checklist-item: yes (adequate method), no (not adequate method), cannot tell (unclear) or not applicable. The complete checklist can be accessed through the original source [16]. NA = not applicable

### Research aim and choice of alternatives

Except for one study [27], a precise aim was reported in all studies including a description of the alternatives, patient population, time horizon and the perspective from which the analysis was conducted (Q1). The alternative treatments were sufficiently described to understand the field of research; and, according to international treatment guidelines [6, 7], the alternatives were meaningful treatments for patients with rCDI (Q2).

### Effectiveness estimates

The application of effectiveness estimates differed between the studies. Whereas some separated the inputs for cure and recurrence of rCDI [24, 28–30], this was not explicitly done in others [25–27] (Additional file 4). Differences in the estimates were also observed between the studies; for example, the probabilities reported for cure ranged between 87.0 and 94.5% for FMT by colonoscopy, 30.8 and 91.6% for oral vancomycin and 42.0 and 93.7% for fidaxomicin. Despite the differences in cure rates, FMT by colonoscopy was associated with the highest probability of cure in all studies. Considerable differences were, however, observed between the studies regarding the order of the probabilities of cure applied for other alternatives.

Generally, the estimates of effectiveness were based on evidence of varying quality including RCTs, cohort studies and case series. Moreover, none of the studies adequately described how the parameter estimates for effectiveness were identified, selected and pooled for the analyses. Only one study included documentation of a pragmatic literature search as a basis for identifying model inputs; however, information on the choice of specific sources and pooling of estimates were not provided [30] (Q3). Apart from one study, which included a head-to-head comparison trial of the two included alternatives [27], all of the health economic evaluations seemed to have applied unadjusted indirect comparisons of the alternatives.

### Cost and consequences

Five studies applied a time horizon of ≤1 year in the analyses [24, 25, 28–30], which limits assessment of all the relevant consequences of treatment alternatives when QALYs are used as the outcome measure. Moreover, all but one study applied utility estimates for rCDI from non-RCDI patient populations, e.g. patients with inflammatory bowel disease or non-infectious diarrhoea, due to lack of evidence on utility estimates for patients with rCDI. The applied utility estimates for rCDI varied greatly from 0.42 to 0.88, whereas the differences between rCDI patients and healthy people ranged from 0.06 to 0.36 (Additional file 4). One study reported a higher utility weight for patients with rCDI than for healthy people [27]. The most recent study applied a utility weight for rCDI based on patients with CDI and a utility weight for the healthy state based on general population norms [30]. This produced a larger difference in utility estimates between rCDI patients and healthy people than in the other articles (Q5/Q6).

The studies reported various perspectives for their analyses. Two of the articles stated that a societal perspective was applied [24, 28]. Nevertheless, in all studies, the costs seemed to be restricted to healthcare costs. Measurement and valuation of costs were generally not reported separately, and parameter estimates were drawn from a variety of sources including national fee schedules, previous research studies and micro-costing based on FMT protocols. Considerable differences were observed between the studies regarding the cost estimates applied for each treatment alternative; for example, the cost ranged between 840 GBP and 3158 GBP for FMT by colonoscopy, 203 GBP and 515 GBP for oral vancomycin, and 1368 GBP and 3155 GBP for fidaxomicin. For FMT, the cost-estimates were based on a variety of assumptions regarding the resource use associated with the treatment. Oral vancomycin or pulse-taper vancomycin was the least costly treatment alternative in most of the studies (*n* = 5) [24, 26–28, 30], whereas FMT by colonoscopy was the least costly in one study [25], and FMT by capsules was the least costly in another study [29]. The most expensive treatment was either fidaxomicin (*n* = 2) [24, 29], FMT by colonoscopy (*n* = 3) [27, 28, 30], FMT by enema (*n* = 1) [26] or pulse-taper vancomycin (n = 1) [25]. The inclusion of the risks and costs of adverse events and hospital admissions varied between the studies (Q5/Q6) (Additional file 4). Costs were extrapolated to a specific reference year in each study, and discounting was used to adjust for differential timing if applicable, e.g. in studies with a time horizon exceeding 1 year [26, 27] (Q7).

### Results and sensitivity analyses

In most of the studies, the incremental cost-effectiveness ratio (ICER) was the primary outcome of the analysis. ICERs were calculated if none of the alternatives compared were considered dominant. In one study, however, an ICER was calculated between FMT by colonoscopy and FMT by oral capsules even though FMT by colonoscopy seemed to be dominant [29] (Q8). All studies applied probabilistic sensitivity analyses, and all but one study included deterministic sensitivity analyses to characterise parameter uncertainties [27]. Examination of methodological and structural uncertainty or heterogeneity was limited. In addition, the studies generally did not provide sufficient descriptions and justifications for the choice of estimates used in the sensitivity analyses. High probabilities that the FMT alternatives were cost-effective compared with antibiotics were found in the probabilistic sensitivity analyses [25–30], but the choice of delivery method was associated with some uncertainty [26–30] (Q9). Lastly, the studies varied with regards to their discussion of study limitations, generalisability and the need for future research (Q10).

## Discussion

In this study, we found that FMT was considered cost-effective in all of the included health economic evaluations. FMT was both cost-saving and more effective than antibiotics in five of the seven identified studies. Although the conclusions varied concerning the most cost-effective delivery method of FMT in all but one study, other delivery methods were still cost effective compared with antibiotic treatments if the preferred method of delivery was unavailable.

The study characteristics differed with regard to analytic method, time horizon, perspective, patient population, number of previous rCDIs, treatment alternatives and the willingness-to-pay threshold applied in the analyses. We identified several methodological concerns that may potentially impact the conclusions of the studies: the choice of effectiveness estimates, the time horizon used in the analysis and the selection of utility and cost estimates.

The reporting of methods used to identify, select and pool parameter estimates for effectiveness was sparse. Without this information, it becomes difficult to judge the validity and applicability of the data included in the analyses and to determine how any biases may impact the results [20]. In this systematic review, we found that the effectiveness measures were based on mixed quality evidence and unadjusted indirect comparisons, possibly resulting in biased estimates. Nonetheless, in all studies, at least one of the FMT delivery methods was associated with a higher probability of cure than all of the antibiotic treatment alternatives. This is in accordance with three out of four RCTs investigating the efficacy of FMT compared with vancomycin [12, 13] and fidaxomicin [14], in which a substantial benefit of FMT (> 39 percentage points) was identified, thereby supporting the validity of the findings of FMT being a cost-effective alternative.

Five studies applied a relatively short time-horizon of ≤1 year in their analyses [24, 25, 28–30]. In general, the time horizon should be sufficiently long to capture all differences in cost and effects [16]. Studies have found that patients with CDI have a significantly higher mortality risk than patients without CDI [31, 32]. Because FMT is expected to have a higher probability of cure than alternative treatment modalities [12–14], patients who receive FMT will, on average, spend less time with CDI than patients treated with other alternatives and thus have a lower risk of dying from CDI. In order to obtain valid cost-effectiveness estimates, this mortality risk difference should be included in the analysis. When QALYs are used as the outcome measure, a short time horizon will most likely not include all of the associated benefits of FMT; and in terms of cost-effectiveness, the results will therefore be biased in favour of antibiotic treatments. Consequently, all other things being equal, FMT may even be more cost-effective than presented in the studies that applied a short time horizon.

The QALY estimate is also affected by the chosen rCDI utility weights. In six studies, the utility weights were drawn from patients with other conditions than rCDI [24–29]. Recent studies have published utility estimates for patients hospitalised with an index or recurrent CDI measured by the EuroQol 5-Dimensions 3-Level (EQ-5D-3L)-questionnaire [33, 34]. The only health economic evaluation applying a CDI-specific utility weight [30] based its input on a UK study by Wilcox et al. [33] in which the authors estimated a utility weight of 0.42 for patients with CDI. A recent French study [34] estimated a utility weight of 0.05 which indicates that substantial variations may exist among patients with CDI. All of these values are significantly lower than the values applied in the remaining health economic evaluations [24–29]. As discussed previously, patients treated with FMT are expected to spend less time with CDI than patients treated with alternatives to FMT. A larger difference in utility weights between healthy patients and patients with CDI would, therefore, lead to FMT being even more cost-effective than concluded in most of the studies. However, a multinational study by Heinrich et al. [35] based on the 36-item Short Form Health Survey (SF-36) found a utility weight of 0.58 and 0.64 in patients with a current and previous CDI episode, respectively. This difference between CDI and “healthy” patients is smaller than the differences applied in most of the health economic evaluations. Though even if this narrow difference was applied, FMT would produce a higher accumulation of QALYs than less effective alternatives and, all other things being equal, FMT would still be the dominant treatment option in the five identified studies in which FMT was associated with lower costs [25–27, 29, 30].

When considering the costs of FMT, the procedure may be divided into three elements: donor recruitment and screening, laboratory processing, and clinical application and follow-up [36]. Currently, these elements vary from one setting to another [37]. This variability may affect both the resource use and effectiveness of FMT, and different processes may, therefore, produce different cost-effectiveness estimates. In some of the studies included in this systematic review, the FMT process was not described in detail, limiting the applicability of the study results in other specific clinical settings. In addition, large variations in the cost estimates for both FMT alternatives and antibiotic treatment alternatives were observed between the health economic evaluations. The cost estimate for a single treatment with the FMT alternatives was higher than for a single treatment with vancomycin in most of the studies. Even so, owing to the increased effectiveness of FMT, which yields a more limited risk of recurrence and its associated costs, all studies concluded that FMT is a cost-effective treatment for rCDI, and some studies reported that FMT may even be cost saving compared with antibiotic treatment alternatives.

In the present study, we performed a systematic literature search in several databases and included methodological quality assessment of the included studies. Quality assessments are subjective evaluations [38] and our results might therefore not be consistent with those of others. Yet, the quality assessments in this study were conducted by two authors who agreed on the final evaluation, and to enhance the transparency of our assessment, we present a detailed quality assessment of each of the studies in Additional file 4.

Future research on the cost-effectiveness of FMT should improve the quality of reporting to aid an assessment of the methodological quality as well as the applicability and generalisability of the analyses. The use of acknowledged methods for identification and synthesis of effectiveness estimates and the use of a life-long time-horizon to capture all relevant costs and consequences would increase confidence in the results of health economic evaluations based on decision analytic modelling. Moreover, there is a need for additional primary research on the health-related quality of life of patients with rCDI to inform future cost-utility analyses.

## Conclusions

FMT is reported as a highly cost-effective treatment alternative compared with antibiotic treatments for patients with rCDI. The identified health economic evaluations varied with respect to many study characteristics and several methodological concerns applied. Considering that recent RCTs have found FMT to be a substantially more effective treatment for rCDI than antibiotics, these concerns do, however, not seem to change the conclusion regarding cost-effectiveness. Still, due to the unstandardised nature of FMT, decision-makers should be aware that the magnitude of costs and effects of FMT may be context specific. Based on the currently available evidence, increased implementation of FMT for rCDI treatment in clinical practice should be strongly considered.

## Supplementary Information

**Additional file 1.** is a .pdf file which contains information on the complete search strategy used in the present systematic review.

**Additional file 2.** is a .pdf file which contains a table with the general study characteristics of the included health economic evaluations.

**Additional file 3.** is a .pdf file which presents an overview of the cost-effectiveness of the different delivery methods of FMT.

**Additional file 4.** is a .pdf file which contains detailed quality assessments of each of the included health economic evaluations.

**Additional file 5.** is a .pdf file which contains a table with the inputs applied in the health economic evaluations.

## Data Availability

Data supporting the conclusions of this article are included within the article and in additional files.

## Abbreviations

CDI: *Clostridioides difficile* infection
rCDI: recurrent *Clostridioides difficile* infection
FMT: Faecal microbiota transplantation
RCT: Randomised controlled trial
GBP: Great Britain pound sterling
QALY: Quality-adjusted life-years
ICER: Incremental cost-effectiveness ratio
EQ-5D-3L: EuroQol 5-Dimensions 3-Level
SF-36: 36-Item Short Form Health Survey
