# Supplementary material for: Health economic evaluations comparing faecal microbiota transplantation with antibiotics for treatment of recurrent Clostridioides difficile infection: a systematic review
Source: Health Econ Rev. 2021 Jan 13;11:3. doi: 10.1186/s13561-021-00301-7 (PMC7805077; doi:10.1186/s13561-021-00301-7)
Supplement: Supplementary file 1 — Additional file 1. is a .pdf file which contains information on the complete search strategy used in the present systematic review. [file 13561_2021_301_MOESM1_ESM.pdf]

Additional file 1: Search strategy

PubMed: ((“Clostridium Infections”[MeSH]) OR (clostridium difficile infection)) AND ((“Fecal Microbiota Transplantation”[MeSH]) OR (fecal microbiota transplantation)) AND ((“Cost-Benefit Analysis”[MeSH]) OR (economic evaluation) OR (cost-benefit) OR (cost-utility) OR (cost-effectiveness))

Embase: ((fecal AND microbiota AND transplantation) OR (fecal microbiota transplantation’/exp)) AND ((clostridium difficile infection’/exp) OR (clostridium AND difficile AND infection)) AND ((economic evaluation’exp) OR (economic AND evaluation) OR (‘cost benefit’ AND analysis) OR (‘cost utility’ AND analysis) OR (‘cost effectiveness’ AND analysis))

Cochrane Library: ((“Clostridium Infections”[MeSH]) OR (clostridium difficile infection)) AND ((“Fecal Microbiota Transplantation”[MeSH]) OR (fecal microbiota transplantation)) AND ((“Cost-Benefit Analysis”[MeSH]) OR (economic evaluation) OR (cost-benefit) OR (cost-utility) OR (cost-effectiveness))

Cinahl: ((MH “Clostridium infections+”) OR (clostridium difficile infection)) AND ((fecal microbiota transplant) OR (fecal microbiota transplantation) OR (intestinal microbiota transfer) OR (intestinal microbiota transfers) OR (fecal transplant) OR (fecal transplantation)) AND ((economic evaluation) OR (cost utility) OR (cost benefit) OR (cost-effectiveness))

Scopus: ((“clostridium difficile infection”) OR (“clostridium difficile”)) AND ((“fecal microbiota transplant”) OR (“intestinal microbiota transfer”) OR (“fecal transplant”) OR (“fecal transplantation”)) AND ((“economic evaluation”) OR (“cost-benefit”) OR (“cost-utility”) OR (“cost-effectiveness”)

EconLit: ((MAINSUBJECT.EXACT("Allocative Efficiency; Cost-Benefit Analysis (D61)") OR (cost-benefit) OR (cost-utility) OR (cost-effectiveness) OR ("economic evaluation")) AND (("clostridium difficile infection") OR (clostridium difficile infection) OR (clostridium difficile)) AND (("fecal microbiota transplant") OR (fecal microbiota transplant) OR (intestinal microbiota transplantation) OR (intestinal microbiota transfer) OR (intestinal microbiota transfers) OR (fecal transplant) OR (fecal transplantation))

NHS Economic Evaluation Database: ((“Clostridium Infections”[MeSH]) OR (clostridium difficile infection)) AND ((“Fecal Microbiota Transplantation”[MeSH]) OR (fecal microbiota transplantation)) AND ((“Cost-Benefit Analysis”[MeSH]) OR (economic evaluation) OR (cost-benefit) OR (cost-utility) OR (cost-effectiveness))
