# Supplementary material for: Health economic evaluations comparing faecal microbiota transplantation with antibiotics for treatment of recurrent Clostridioides difficile infection: a systematic review
Source: Health Econ Rev. 2021 Jan 13;11:3. doi: 10.1186/s13561-021-00301-7 (PMC7805077; doi:10.1186/s13561-021-00301-7)
Supplement: Supplementary file 2 — Additional file 2. is a .pdf file which contains a table with the general study characteristics of the included health economic evaluations. [file 13561_2021_301_MOESM2_ESM.pdf]

Additional file 2: Study characteristics

The table includes the general study characteristics of the health economic evaluations included in the study. QALYs = quality-adjusted life years. rCDI = recurrent *Clostridioides difficile* infection. FMT= faecal microbiota transplantation.

**Author and year**	**Konijeti et al., 2014 [1]**	**Varier et al., 2015 [2]**	**Lapointe-Shaw et al., 2016 [3]**	**Merlo et al., 2016 [4]**	**Baro et al., 2017 [5]**	**Luo et al., 2020 [6]**	**Abdali et al., 2020 [7]**
**Setting/country**	United States	United States	Canada	Australia	France	United States	United Kingdom
**Analytic method**	Decision-tree	Decision-tree	Markov, 6-weeks cycle	Markov, 10-days cycle	Decision-tree	Decision-tree	Markov, 2-months cycle
**Time horizon**	1 year, up to 2 additional recurrences	90 days, up to 1 additional recurrence	18 weeks for costs, lifetime for effects, up to 2 additional recurrences	Not reported - presumably lifetime, number of additional recurrences not reported	78 days, up to 1 additional recurrence	6 months, up to 2 additional recurrences	1 year, up to 2 additional recurrences
**Perspective**	Societal	Third-party payer	Healthcare system	Not reported - presumably societal	Societal	Modified third-party payer	Healthcare system
**Outcome measure**	QALYs	QALYs	QALYs	QALYs, life years	QALYs	QALYs	QALYs
**Patient population at inclusion**	First rCDI, outpatient, mild-to-moderate	Third rCDI, outpatient, severity not reported	First rCDI, severity not reported	≥1 rCDI, severity not reported	Second rCDI, outpatient, mild-to-moderate	First rCDI, outpatient, mild-to-moderate,	≥1 rCDI, hospital-lised, severity not reported
**Intervention**	FMT colonoscopy, enema or duodenal infusion.	FMT colonoscopy	FMT colonoscopy, enema, or nasogastric tube	FMT colonoscopy or nasoduodenal tube	FMT colonoscopy, enema, or duodenal infusion	FMT colonoscopy or capsules	FMT colonoscopy or nasogastric tube
Preceding treatment	Oral vancomycin 500mg qid x 4d	Not reported	Oral vancomycin 125xmg qid x 14d	Oral vancomycin 125mg qid x 4-5d and bowel lavage	Oral vancomycin 500mg qid x 4d	Oral vancomycin 125mg qid x 10d	Not reported
Treatment in case of additional recurrences	FMT similar delivery method (second recurrence), oral pulse-taper vancomycin 125 mg qid x 10d + 125 mg every third day x 10 doses (third recurrence)	Oral vancomycin 250 mg qid x 14d + 6 weeks oral vancomycin taper	FMT similar delivery method	FMT similar delivery method (first subsequent recurrence), oral vancomycin 125mg qid x 10d (additional recurrences)	FMT similar delivery method (mild-to-moderate or severe uncomplicated rCDI), oral vancomycin 500mg qid x10d plus intravenous metronidazole 500mg tid x10d (severe complicated rCDI)	FMT similar delivery method	FMT similar delivery method (first subsequent recurrence), oral vancomycin 6-weeks taper pulse starting with 250mg (second subsequent recurrence)

**Control treatment**<br><br>Treatment in case of additional recurrences	Oral metronidazole 500mg tid x 10d, oral vancomycin 125mg qid x 10d, or oral fidaxomicin 200mg bid x 10d<br><br>Patients treated with metronidazole: oral vancomycin (second recurrence) or pulse-taper vancomycin (third recurrence)<br>Patients treated with vancomycin or fidaxomicin: pulse-taper vancomycin (second recurrence) or FMT by colonoscopy (third recurrence)	Oral vancomycin 250 mg qid x 14d + 6 weeks oral vancomycin taper<br><br>Oral vancomycin 250 mg qid x 14d + 6 weeks oral vancomycin taper	Oral metronidazole 500mg tid x 14d, oral vancomycin 125mg qid x 14d, or oral fidaxomicin 200mg bid x 10d<br><br>6 weeks taper-pulse oral vancomycin: 125 mg qid x 14d + 125 mg bid x 7d + 125mg qd x 7d + 125 mg qod x 7d + 125 mg every third day x 7d	Oral vancomycin 125mg qid x 14d<br><br>Oral vancomycin 125mg qid x 10d	Pulsed-tapered vancomycin 125mg qid x 10d + 500mg qod x 21d, or fidaxomicin 200mg tid x 10d<br><br>Similar treatment (mild-to-moderate or severe uncomplicated rCDI) or oral vancomycin plus intravenous metronidazole (severe complicated rCDI)	Tapered vancomycin 125 mg qid x 14d + 125 mg bid x 7d + 125 mg qd x 7d + 125 mg qod x 7d + 125 mg every third day x 7d, fidaxomicin 200mg bid x10d, or a combination of bezlotoxumab 10mg/kg one-time infusion and oral vancomycin 125mg qid x 10d<br><br>Not reported (second recurrence), FMT by colonoscopy (third recurrence).	Oral vancomycin 250 or 500mg qid x 10d or oral fidaxomicin 200mg x 10d<br><br>Similar treatment for vancomycin (first subsequent recurrence), oral vancomycin 6-weeks taper pulse starting with 250mg (second subsequent recurrence)
**WTP threshold** (2019-level GBP)	37,860 GBP (50,000 USD, 2012-level)	Not reported	30,923 GBP (50,000 CAN, 2014-level)	Not reported	30913 GBP (32,000 EUR, 2016-level)	68,000 GBP (100,000 USD, 2019-level)	20,340 GBP (20,000 GBP, 2018-level)
**Cost-effective alternative** (2019-level GBP)	FMT colonoscopy more effective and costlier than vancomycin, ICER 12,884 GBP/QALY (17,106 USD, 2012-level)	FMT colonoscopy more effective and less costly (dominant) than vancomycin.	FMT colonoscopy more effective and less costly (dominant) than vancomycin	FMT nasoduodenal tube and FMT colonoscopy more effective and less costly (dominant) than vancomycin[a].	FMT enema more effective and costlier than pulsed-tapered vancomycin, ICER 17,478 GBP/QALY (18,092 EUR, 2016-level)	FMT colonoscopy and FMT capsules more (or equally) effective and less costly (dominant) than all other alternatives[b].	FMT nasogastric tube (dominant compared with antibiotics). FMT colonoscopy more effective and costlier than FMT nasogastric tube ICER 246,642 GBP/QALY (242,524 GBP, 2018-level)

[a]FMT by nasoduodenal tube is reported as being equally effective but less costly than FMT by colonoscopy. FMT by nasoduodenal tube is therefore considered the most cost-effective alternative by the authors of the present systematic review.

[b]FMT by colonoscopy is reported as being more effective and less costly than all other alternatives (Table 3 in Luo et al.). FMT by colonoscopy is therefore considered the most cost-effective alternative by the authors of the present systematic review.

References

1. Konijeti GG, Sauk J, Shrime MG, Gupta M, Ananthakrishnan AN. Cost-effectiveness of competing strategies for management of recurrent clostridium difficile infection: A decision analysis. Clinical Infectious Diseases. 2014;58(11):1507-1514.
2. Varier RU, Biltaji E, Smith KJ, et al. Cost-Effectiveness Analysis of Fecal Microbiota Transplantation for Recurrent Clostridium difficile Infection. Infection Control and Hospital Epidemiology. 2015;36(4):438-444.
3. Lapointe-Shaw L, Tran KL, Coyte PC, et al. Cost-Effectiveness Analysis of Six Strategies to Treat Recurrent Clostridium difficile Infection. PloS one. 2016;11(2):e0149521.
4. Merlo G, Graves N, Brain D, Connelly LB. Economic evaluation of fecal microbiota transplantation for the treatment of recurrent Clostridium difficile infection in Australia. Journal of gastroenterology and hepatology. 2016;31(12):1927-1932.
5. Baro E, Galperine T, Denies F, et al. Cost-Effectiveness Analysis of Five Competing Strategies for the Management of Multiple Recurrent Community-Onset Clostridium difficile Infection in France. PloS one. 2017;12(1):e0170258.
6. Luo Y, Lucas AL, Grinspan AM. Fecal Transplants by Colonoscopy and Capsules Are Cost-Effective Strategies for Treating Recurrent Clostridioides difficile Infection. Digestive diseases and sciences. 2020;65(4):1125-1133.
7. Abdali ZI, Roberts TE, Barton P, Hawkey PM. Economic evaluation of Faecal microbiota transplantation compared to antibiotics for the treatment of recurrent Clostridioides difficile infection. EClinicalMedicine. 2020;24:100420.
