# Supplementary material for: Health economic evaluations comparing faecal microbiota transplantation with antibiotics for treatment of recurrent Clostridioides difficile infection: a systematic review
Source: Health Econ Rev. 2021 Jan 13;11:3. doi: 10.1186/s13561-021-00301-7 (PMC7805077; doi:10.1186/s13561-021-00301-7)
Supplement: Supplementary file 3 — Additional file 3. is a .pdf file which presents an overview of the cost-effectiveness of the different delivery methods of FMT. [file 13561_2021_301_MOESM3_ESM.pdf]

Additional file 3: Assessment of cost-effectiveness

The table includes an assessment of the cost-effectiveness of faecal microbiota transplantation (FMT) compared with antibiotics in alternative treatment scenarios. If indicated, an incremental cost-effectiveness ratio (ICER) is calculated as costs per additional quality-adjusted life-year (QALY) between the relevant alternatives. The ICER is compared with the willingness-to-pay (WTP)-threshold stated in the study: if the ICER is below the WTP-threshold, the alternative is considered cost-effective. The cost-effective treatment alternative in each scenario is indicated in italics. Authors' calculation/interpretation refer to calculations/interpretations made by the authors of the present systematic review.

**Konijeti et al., 2014 [1], (WTP=50,000)**			
**Scenario: All strategies available (as reported in the study)**			
**Treatment**	**Costs**	**QALYs**	**ICER**
Vancomycin	2,912	0.8580	
FMT colonoscopy	*3,149*	*0.8719*	*17,016*
Metronidazole	3,941	0.8292	Dominated
FMT enema	4,090	0.8543	Dominated
FMT duodenal infusion	4,208	0.8553	Dominated
Fidaxomicin	4,261	0.8653	Dominated
**Scenario: Restricted to FMT enema (as reported in the study)**			
**Treatment**	**Costs**	**QALYs**	**ICER**
Vancomycin	*3,488*	*0,8485*	
Metronidazole	3,941	0,8292	Dominated
FMT enema	4,090	0,8543	105,003
Fidaxomicin	4,602	0,8597	99,862
**Scenario: Restricted to FMT duodenal infusion (as reported in the study)**			
**Treatment**	**Costs**	**QALYs**	**ICER**
Vancomycin	*3,531*	*0.8484*	
Metronidazole	3,941	0.8292	Dominated
FMT duodenal infusion	4,208	0.8553	97,352
Fidaxomicin	4,628	0.8596	98,443

**Varier et al., 2015 [2], (WTP=not reported)**			
**Scenario: All strategies available (as reported in the study)**			
**Treatment**	**Costs**	**QALYs**	**ICER**
FMT colonoscopy	*1669*	*0.242*	
Vancomycin	3788	0.235	Dominated

**Lapointe-Shaw et al., 2016 [3], (WTP=50,000)**			
**Scenario: All strategies available (as reported in the study)**			
**Treatment**	**Costs**	**QALYs**	**ICER**
FMT colonoscopy	*5,246*	*9.40*	
Metronidazole	5,386	9.09	Dominated
FMT enema	5,667	9.26	Dominated
Vancomycin	5,929	9.03	Dominated
FMT nasogastric tube	5,935	9.15	Dominated
Fidaxomicin	7,319	9.16	Dominates
**Scenario: Restricted to FMT enema (as reported in the study)**			
**Treatment**	**Costs**	**QALYs**	**ICER**
Metronidazole	5,386	9.09	
FMT enema	*5,667*	*9.26*	*1,708*
Vancomycin	5,929	9.03	Dominated
Fidaxomicin	7,319	9.16	Dominated
**Scenario: Restricted to FMT nasogastric tube (authors' calculations)**			
**Treatment**	**Costs**	**QALYs**	**ICER**
Metronidazole	5,386	9.09	
Vancomycin	5,929	9.03	Dominated
FMT nasogastric tube	*5,935*	*9.15*	*9,150*
Fidaxomicin	7,319	9.16	138,400

**Merlo et al., 2016 [4], (WTP=not reported)**			
**Scenario: All strategies available (as reported in the study)**			
**Treatment**	**Incremental costs compared with vancomycin**	**Incremental QALYs compared with vancomycin**	**ICER**
FMT nasoduodenal tube	*-4,094*	*1.2*	
FMT colonoscopy	-4,045	1.2	Dominated (authors' interpretation)
Vancomycin			Dominated
**Scenario: Restricted to FMT colonoscopy (authors' calculations)**			
**Treatment**	**Incremental costs compared with vancomycin**	**Incremental QALYs**	**ICER**
FMT colonoscopy	*-4,045*	*1.2*	
Vancomycin			Dominated

**Baro et al., 2017 [5], (WTP=32,000)**			
**Scenario: All strategies available (as reported in the study)**			
**Treatment**	**Costs**	**QALYs**	**ICER**
Vancomycin	1,235	0.1812	
FMT enema	*1,610*	*0.2019*	*18,092*
FMT colonoscopy	1,816	0.2047	73,571
FMT duodenal infusion	1,834	0.2013	Dominated
Fidaxomicin	2,464	0.1988	Dominated
**Scenario: Restricted to FMT colonoscopy (authors' calculations)**			
**Treatment**	**Costs**	**QALYs**	**ICER**
Vancomycin	1,235	0.1812	
FMT colonoscopy	*1,816*	*0.2047*	*24,723*
Fidaxomicin	2,464	0.1988	Dominated
**Scenario: Restricted to FMT duodenal tube (authors' calculations)**			
**Treatment**	**Costs**	**QALYs**	**ICER**
Vancomycin	1,235	0.1812	
FMT duodenal infusion	*1,834*	*0.2012*	*29,801*
Fidaxomicin	2,464	0,1988	Dominated

**Luo et al., 2020 [6], (WTP=100,000)**			
**Scenario: All strategies available (as reported in the study)**			
**Treatment**	**Costs**	**QALYs**	**ICER**
FMT colonoscopy	*5,250*	*0.435*	
FMT capsules	5,436	0.429	Dominated (authors' interpretation)
Vancomycin	7,006	0.421	Dominated
Fidaxomicin	7,557	0.429	Dominated
Bezlotoxumab	9,612	0.426	Dominated
**Scenario: Restricted to FMT capsules (authors' calculations)**			
**Treatment**	**Costs**	**QALYs**	**ICER**
FMT capsules	*5,436*	*0.429*	
Vancomycin	7,006	0.421	Dominated
Fidaxomicin	7,557	0.429	Dominated
Bezlotoxumab	9,612	0.426	Dominated

**Abdali et al., 2020 [7], (WTP=20,000)**			
**Scenario: All strategies available (as reported in the study)**			
**Treatment**	**Costs**	**QALYs**	**ICER**
FMT nasogastric tube	*8,877*	*0.645*	
FMT colonoscopy	11,716	0.657	24,2514
Fidaxomicin	14,339	0.577	Dominated
Vancomycin	17,279	0.513	Dominated

Scenario: Restricted to FMT colonoscopy (authors' calculations)			
**Treatment**	**Costs**	**QALYs**	**ICER**
FMT colonoscopy	*11,716*	*0.657*	
Fidaxomicin	14,399	0.577	Dominated
Vancomycin	1,729	0.513	Dominated

References

1. Konijeti GG, Sauk J, Shrime MG, Gupta M, Ananthakrishnan AN. Cost-effectiveness of competing strategies for management of recurrent clostridium difficile infection: A decision analysis. Clinical Infectious Diseases. 2014;58(11):1507-1514.
2. Varier RU, Biltaji E, Smith KJ, et al. Cost-Effectiveness Analysis of Fecal Microbiota Transplantation for Recurrent Clostridium difficile Infection. Infection Control and Hospital Epidemiology. 2015;36(4):438-444.
3. Lapointe-Shaw L, Tran KL, Coyte PC, et al. Cost-Effectiveness Analysis of Six Strategies to Treat Recurrent Clostridium difficile Infection. PloS one. 2016;11(2):e0149521.
4. Merlo G, Graves N, Brain D, Connelly LB. Economic evaluation of fecal microbiota transplantation for the treatment of recurrent Clostridium difficile infection in Australia. Journal of gastroenterology and hepatology. 2016;31(12):1927-1932.
5. Baro E, Galperine T, Denies F, et al. Cost-Effectiveness Analysis of Five Competing Strategies for the Management of Multiple Recurrent Community-Onset Clostridium difficile Infection in France. PloS one. 2017;12(1):e0170258.
6. Luo Y, Lucas AL, Grinspan AM. Fecal Transplants by Colonoscopy and Capsules Are Cost-Effective Strategies for Treating Recurrent Clostridioides difficile Infection. Digestive diseases and sciences. 2020;65(4):1125-1133.
7. Abdali ZI, Roberts TE, Barton P, Hawkey PM. Economic evaluation of Faecal microbiota transplantation compared to antibiotics for the treatment of recurrent Clostridioides difficile infection. EClinicalMedicine. 2020;24:100420.
