# Supplementary material for: Health economic evaluations comparing faecal microbiota transplantation with antibiotics for treatment of recurrent Clostridioides difficile infection: a systematic review
Source: Health Econ Rev. 2021 Jan 13;11:3. doi: 10.1186/s13561-021-00301-7 (PMC7805077; doi:10.1186/s13561-021-00301-7)
Supplement: Supplementary file 4 — Additional file 4. is a .pdf file which contains detailed quality assessments of each of the included health economic evaluations. [file 13561_2021_301_MOESM4_ESM.pdf]

Additional file 4: Individual methodological quality assessments

Results of the quality assessment of each individual health economic evaluation included in the study [1-7]. The items in the table are based on the checklist for assessing economic evaluations reported by Drummond et al., 2015 [8].

Section/item	Konijeti et al., 2014 [1]
**Question 1.**	**Yes.** The study examines both the costs and effects of several alternative treatments for rCDI. The study is based on a relatively short time horizon of one year. The study states that a societal perspective is applied in the analysis. The patient population is described adequately. No subgroups are defined.
**Question 2.**	**Yes.** The alternatives are relevant and described to a reasonable extent. The pharmacological treatments are described in a degree to which replication of the procedure is possible. The description of FMT is adequate for understanding the research question.
**Question 3.**	**Cannot tell.** The study uses measures of effectiveness from several different sources. However, although the sources are referenced, there is no description of how these studies were identified, selected and pooled for the analysis. Both randomised controlled trials and small observational studies are included in the analysis, but no discussion on the risk of bias is included. The limited transparency makes it difficult to judge the validity of the estimates.
**Question 4.**	**No.** There seems to be a problem with the choice of the model structure and reported perspective. The analysis is conducted in TreeAge using a decision-tree framework; however, the authors want to estimate the number of quality-adjusted life-years gained, defined as time spent in different health states. Thus, it would have made more sense to use a Markov model structure. Moreover, the full numbers of QALYs gained are not included because of the short time horizon. The study seems to include relevant costs for the different alternatives; costs related to the pharmaceuticals, FMT laboratory testing, preparation, and installation, outpatient visits, and hospitalisation. The risks of adverse events are not included in the study. In the study, it is stated that a societal perspective is used for the analysis. However, the analysis seems to be limited to healthcare-related costs.
**Question 5.**	**Cannot tell.** The study does not separate the measurement and valuation of costs and consequences.
**Question 6.**	**Cannot tell.** The sources for costs and consequences are referenced but not described except for items related to FMT. The study applies the Center for Medicare and Medicaid Services (CMS) fees for most of the items. Concerning the consequences of different alternatives, it is not apparent how the QALY calculations are performed in the tree. The study uses utility measures from patients with ulcerative colitis due to lack of evidence on patients with rCDI. It is not described how these patients relate to patients with rCDI.
**Question 7.**	**Not applicable.** The study is reported in 2012 USD. The time horizon of the study is one year, why discounting is not applicable. This is, however, not mentioned in the study.
**Question 8.**	**Yes.** The cost and consequences of the different alternatives are appropriately compared in incremental analyses.
**Question 9.**	**Yes.** The study includes both deterministic and probabilistic sensitivity analyses. Sufficient justifications on the choice of ranges are not provided for the probabilistic sensitivity analysis. In this regard, there are also no justification for using a triangular distribution for utilities. The results from the probabilistic sensitivity analysis are only represented as an ICER-value, and the decision uncertainty is not reflected as the probability of the alternatives being cost-effective at certain thresholds. The deterministic sensitivity analyses consist of one-way threshold analyses. The entire possible range is included for probabilities and utilities, and broad ranges are included for costs. The study conclusions are sensitive to changes in the probabilities of cure and recurrence for vancomycin and FMT by colonoscopy. However, the cure rate for vancomycin should exceed the cure rate of FMT to change the conclusion. Also, relatively large changes in the costs of colonoscopy, fidaxomicin and vancomycin could affect the conclusions.
**Question 10.**	**Cannot tell.** The conclusions are based on the results of the analyses, and the impact of some uncertainties are acknowledged. However, there is no discussion on the impact of any possible biases from the use of small case series to inform the effectiveness of FMT and the effect of the relatively short time horizon applied in the study. In addition, the generalizability of the results is not discussed. There is no comparison with existing economic evaluations within the area; however; this seems reasonable, as the study is the first of its kind. The study does, however, discuss several issues of concern to the user of the economic evaluation, including changes in the FMT procedure and start-up costs, the possibility of heterogeneity related to strains and recurrence rates. The need for future research in treatment effectiveness is mentioned briefly.

Section/item	Varier et al., 2015 [2]
**Question 1.**	**Yes.** The study examines both the costs and effects of the alternative treatments for patients with rCDI from a third-party payer perspective. The time horizon is relatively short (90 days). The patient population is described adequately, but more information, e.g. on the severity, could have been provided. No subgroups are defined for the analysis.
**Question 2.**	**Yes.** The alternatives included in the analysis are relevant. Other alternatives could have been included in the analysis, e.g. fidaxomicin. The grounds for omission are described as being due to limited evidence, but this is not thoroughly discussed (the evidence regarding FMT was also limited at the time but still included in the study).

**Question 3.**	**Cannot tell.** The study uses several different sources to inform on the effectiveness of alternative treatments. However, there is no description of how these studies were identified, selected, and pooled for the analysis. Studies of different quality are included in the trial, including studies with observational study designs for FMT. This is acknowledged, but the impact is not further discussed. The limited transparency makes it difficult to judge the validity of the estimates.
**Question 4.**	**No.** The study applies a decision-tree model and a short time-horizon. In addition, CDI-related mortality is not included in the analysis. This limits the estimation of the full numbers of QALYs gained from more effective treatments. It is not clear how the QALY calculations are performed in the tree. The study uses a third-party payer perspective for the analysis. The inclusion of healthcare-related costs seems reasonable. The cost of FMT includes both donor and recipient screening, the procedure and facility costs. However, the description of FMT could have been elaborated to enhance the applicability and generalizability of the results. Yet, the cost of adverse events from the procedure was included.
**Question 5.**	**Cannot tell.** The study does not separate the measurement and valuation of costs and consequences.
**Question 6.**	**Cannot tell.** The study refers to the sources, but do not adequately justify the inclusion of studies. The study uses the Center for Medicare and Medicaid Services (CMS) fees and costs from previously published sources. Concerning the consequences of different alternatives, the study uses utility measures from patients with other diseases (e.g. non-infectious diarrhoea) due to lack of evidence on patients with rCDI. It is not discussed how these patients relate to patients with rCDI. Moreover, the utility value for healthy individuals is set to 1, which seems high, considering the patient population at risk of rCDI.
**Question 7.**	**Not applicable.** Costs are estimated in 2011 USD. The study uses a time horizon of 90 days. Therefore, discounting is not relevant to the analysis. This is mentioned in the article.
**Question 8.**	**Yes.** No incremental analysis was done as FMT was the dominant alternative (i.e. associated with larger effects and lower costs) compared with vancomycin. This seems reasonable.
**Question 9.**	**Yes.** The uncertainty of the parameter estimates was investigated in deterministic and probabilistic sensitivity analyses. However, justifications were not sufficiently provided. Deterministic sensitivity analyses identified parameter thresholds (e.g. for FMT cost and probabilities of cure with FMT or vancomycin) that could change the conclusions. Still, no discussion on the likelihood of the thresholds was given. The probabilistic sensitivity analysis showed a 100 % probability of FMT being the dominant (cost-effective) alternative compared with vancomycin.
**Question 10.**	**Cannot tell.** The conclusions were based on the results of the primary analysis and sensitivity analyses. The results were compared to the one existing economic evaluation within the area, and differences in study design were acknowledged and discussed. Also, two other studies investigating first-line treatments for CDI were mentioned. The study did not sufficiently address the generalizability of the study but discussed several limitations of the study and the need for further research within the area. However, several limitations were not adequately considered, including the quality of evidence, utility measures, and omission of fidaxomicin.

**Section/item**	**Lapointe-Shaw et al., 2016 [3]**
**Question 1.**	**Yes.** The study examines both the costs and effects of different alternatives for the treatment of patients with rCDI. The time horizon is relatively short for costs (18 weeks) while the time horizon for outcomes is the remaining lifetime. The relatively short time horizon for costs is described as being due to the uncertain effectiveness of alternatives over time (limited available evidence). The study uses the perspective of the Ontario Ministry of Health and Long-term Care in Canada for the analysis. The patient population is adequately described for the analysis. No subgroups are defined.
**Question 2.**	**Yes.** The study includes all relevant alternatives. The competing alternatives are described in sufficient detail to judge the applicability of the results.
**Question 3.**	**Cannot tell.** The study uses several different data sources to inform on the effectiveness of the alternative treatments. All sources are referenced, and a description is given on the pooling of data. The probabilities for FMT are based on studies identified in another systematic literature review and an RCT, while the probabilities for vancomycin is estimated based on a secondary analysis of an RCT. Besides being referenced, the data used for the other alternatives are not described sufficiently.
**Question 4.**	**Yes.** The analysis seems to be a well-structured. The study uses a Markov model with a lifetime horizon. The study uses a perspective of the Ontario Ministry of Health and Long-term Care for the analysis in Canada. The study notes that the cost of hospital care, physician services and a proportion of drug costs are incurred from this perspective. The relevant costs for this perspective seem to have been identified. For FMT this entails costs of laboratory testing/screening, capital costs and costs of the procedure. Adverse events i.e. the risk of death from colonoscopy or nasogastric tube are included.
**Question 5.**	**Yes.** The study does not separate the measurement and valuation of costs and consequences.
**Question 6.**	**Yes.** The costs are drawn from several separate sources, including published literature, estimates from hospitals and local databases. The study included a short description of the source and assumptions for each cost item, which

	enhances the transparency. The utility measures are from different patient populations than patients with rCDI, and the methods used for measurement and valuation are not reported. However, it is acknowledged that the estimate might be different for patients with rCDI, and the estimates are varied in sensitivity analyses.
**Question 7.**	**Yes.** The costs are estimates in 2014 CAD, and a consumer price index is used for inflation if necessary. Costs are not discounted due to the time horizon being less than one year. However, outcomes (QALYs) are calculated over the remaining lifetime. A discount rate of 5 % is applied and justified by local recommendations.
**Question 8.**	**Yes.** Incremental analysis of costs and consequences is performed. Still, the results are not presented as an incremental cost-effectiveness ratio (ICER) as FMT are estimated to be both less costly and more effective than the alternatives (i.e. dominant). This is reasonable. ICERs are presented in scenario analyses where indicated.
**Question 9.**	**Yes.** Both deterministic and probabilistic sensitivity analyses are applied to examine the parameter uncertainty. Ranges are calculated or specified by the authors. More information on the choices could have been provided. The conclusion is sensitive to the estimated recurrence rate after FMT with an enema. Moreover, several scenario analyses were performed, and the choice of the discount rate was examined, but it did not change the conclusions. The probabilistic sensitivity illustrated 87% probability of FMT being cost-effective at the stated WTP-threshold.
**Question 10.**	**Yes.** The conclusions of the study are based on the results of the analysis. The authors acknowledge the uncertainties about the decision and the limited quality of input parameters. The results were compared to one existing health economic evaluation, and differences in conclusions and study design were discussed. In addition, the generalizability of the study is discussed briefly, and other central factors for the decision is highlighted, such as the lack of facilities, institutional support, and upfront costs. Implications for future research are not explicitly highlighted.

**Section/item**	**Merlo et al., 2016 [4]**
**Question 1.**	**No.** The study compares the costs and outcomes of different alternative treatments for patients with rCDI. The time horizon is not explicitly reported, but descriptions indicate a life-long time horizon. Additionally, the perspective for the analysis is not stated, but other descriptions indicate a societal perspective was the basis for the analysis. The descriptions of the population are adequate. No subgroups are identified.
**Question 2.**	**Yes.** The study included three relevant alternatives. The study could have included fidaxomicin in the analysis. This is not acknowledged. A comprehensive description is given on the included alternatives which aid the applicability and possibility to replicate the study.
**Question 3.**	**Cannot tell.** The study includes a randomised controlled trial comparing FMT via nasoduodenal tube and vancomycin to inform on the effectiveness of the different treatment modalities. The study is based on a relatively small number of patients (n=42), and the dosage of vancomycin differs from what is applied in the economic evaluation. Also, the effectiveness of the different delivery methods of FMT is considered to be equal based on a small randomised pilot study (n=20). These limitations are not discussed. Several sources are used to inform on other outcomes, but the selection of the studies is not described.
**Question 4.**	**Cannot tell.** The study uses a Markov model. However, the study did not explicitly specify the perspective and time-horizon of the study, causing the evaluation of this item to be challenging. However, the study included relevant healthcare-related costs and consequences. Each input for treatment with FMT is explained in detail including; recruitment, screening, preparation, pre-treatment, application and supervision after the procedure. The study includes a note on the assumption that indirect costs are considered to be included on the utility-measures, indicating that a societal perspective is used for the analyses. However, only healthcare costs seem to have been included, and additional costs for patients or society is not included.
**Question 5.**	**Cannot tell.** The resource use and prices are not reported separately besides for FMT. In addition, the measurement and valuation of consequences are not separated.
**Question 6.**	**Cannot tell.** The sources for costs and consequences were reported, but no specific justifications were made on the choice of sources. Benefit schedules and national cost databases are applied. The study includes two outcome measures; life-years and quality-adjusted life years. However, the utility measures are based on other patient populations than patients with rCDI (patients with diarrhoea related to cancer treatment). This is not acknowledged or included in a sensitivity analysis. The utility values for healthy people are lower than for patients with rCDI, which seems counterintuitive.
**Question 7.**	**Yes.** Costs were reported in 2015 AUD. Both costs and consequences were discounted. The rate was based on national standards.
**Question 8.**	**Yes.** The study described that an incremental analysis of the costs and consequences would be performed. Both types of FMT was found to be equally effective and more effective than vancomycin. Also, both types of FMT were less costly than vancomycin. However, FMT by colonoscopy was insignificantly costlier than FMT by nasoduodenal tube. Therefore, FMT by nasoduodenal tube weakly dominates FMT by colonoscopy. This is not acknowledged in the study.

**Question 9.**	**No.** The study included only a probabilistic sensitivity analysis. No deterministic sensitivity analyses are described. A short description of the ranges is given; however, justifications are not provided. The results of the PSA are presented in figures, which shows a high probability of FMT being cost-effective, but the findings are not described in the study.
**Question 10.**	**No.** The study included a short discussion. The main results are described but not the results from the sensitivity analysis. The results are not compared with those of others, and the generalizability of the results are not discussed. A short description is given on some of the limitations of the study; however, this is not considered to include all issues of concern (e.g. the evidence included, choice of alternatives, etc.). Yet, the study does note some additional concerns about FMT (i.e. upfront costs, regulatory issues and acceptability of FMT).

Section/item	Baro et al., 2017 [5]
**Question 1.**	**Yes.** The study examines both the costs and consequences of different alternative treatments for rCDI. The time horizon applied is relatively short (78 days). The study reports that a societal perspective is used for the analysis. The description of the population was adequate. The study did not include any subgroups.
**Question 2.**	**Yes.** The study compares several relevant alternative treatments for rCDI. No relevant alternatives seem to have been omitted. An adequate description is provided for each of the alternatives.
**Question 3.**	**Cannot tell.** The study includes many different sources to inform on effectiveness for each of the alternatives. The sources are referenced, and descriptions on some of the sources are included. No statements are provided on how the studies were identified and pooled for the analysis. The studies cover both randomised controlled trials and observational studies. This is noted, but the impacts of this are not further discussed. The relative effectiveness applied for enema seems high, possibly explaining the results of the analysis. The limited transparency makes it difficult to judge the validity of the estimates.
**Question 4.**	**No.** The study uses a decision-tree model with a short time-horizon. This limits the estimation of the full benefit of FMT in terms of quality-adjusted life-years. The study claims to apply a societal perspective for the analysis, but the analysis seems to be limited to healthcare-related costs and does not include any indirect costs. Relevant costs and consequences seem to have been identified considering the healthcare-related costs. FMT included screening, preparation, application, outpatient visits and adverse events.
**Question 5.**	**Yes.** The study does not separate the measurement and valuation of costs and consequences.
**Question 6.**	**Yes.** The sources of data are referenced, and descriptions, as well as some justifications, for the use of studies are provided. An adequate description of costs is provided. The study uses QALY's as the effectiveness measure; however, the utility measures are based on EQ-5D estimates drawn from populations with non-infectious diarrhoea. This is acknowledged but not further discussed. Also, the utility value for healthy individuals is set to 1, which seems high, considering the patient population at risk of rCDI. Yet the utilities are varied in a deterministic sensitivity analysis without changing the conclusions.
**Question 7.**	**Not applicable.** Costs are reported in 2016 EUR. The study includes a time horizon of less than a year. Therefore, discounting is not indicated. However, this is not noted in the study.
**Question 8.**	**Yes.** The study includes an incremental analysis of costs and consequences when indicated.
**Question 9.**	**Yes.** The study applies both deterministic and probabilistic sensitivity analyses and scenario analysis. Uncertainties related to the costs of FMT are not specified with the reason of it being unavailable. Therefore, these items are not included in the probabilistic sensitivity analysis. However, a threshold analysis is applied considering the costs of FMT. The univariate sensitivity analyses compare pulse/taper vancomycin with FMT by enema and show that the probability of cure of vancomycin and FMT by enema may influence the conclusions of the study. In the probabilistic sensitivity analysis, FMT by enema had a probability of 58% while FMT by colonoscopy had a probability of 19% of being the most cost-effective alternative at the stated WTP-threshold.
**Question 10.**	**Cannot tell.** The discussion includes a short description of the main results of the base-case analysis. No statement is made on the sensitivity analyses, but these were adequately described in the result-section. The study includes a comparison of study results with three other economic evaluations and briefly mentions areas of variation. The generalizability of the study results or any issues of implementation is not discussed. However, the study notes the possibility of using frozen faeces for FMT but does not point out areas for further research. Some strengths and limitations are mentioned; however, the choice of studies to inform the model could have been elaborated.

Section/item	Luo et al., 2020 [6]
**Question 1.**	**Yes.** The study examines both the cost and consequences of several alternatives for the treatment of rCDI. The time horizon is relatively short (six months). The study applies a modified third-party payer perspective and includes a description of areas covered by this perspective. The patient population is adequately described. No subgroups are included.

**Question 2.**	**Yes.** The study considers several relevant alternatives for the treatment of rCDI. Different delivery methods for FMT exist and could have been included, or a discussion on the omission could have been provided. The alternatives are described in sufficient detail to judge the applicability of the analysis.
**Question 3.**	**Cannot tell.** The study includes several sources to inform on the effectiveness of the different alternatives. The study does not describe how the studies were identified, selected and pooled for the analysis. However, the studies are referenced. The studies included are both randomised controlled trials and observational studies. The possible limitations of using these studies are acknowledged, but the impact is not further discussed. The limited transparency makes it difficult to judge the validity of the estimates.
**Question 4.**	**No.** The study applied a decision-tree model with a time horizon of six months. Thus, the full benefits of a more effective treatment are not included. The study applies a modified third-party payer perspective which included costs related to medication, hospitalisations and relevant procedures. Treatment with FMT consists of the delivery method and the product. The description of the pharmacological treatment consists of the dosage and treatment regimen. The study does not include outpatient visits for follow-up or the risk of adverse events.
**Question 5.**	**Cannot tell.** The measurement and valuation of costs and consequences are not separately reported.
**Question 6.**	**Cannot tell.** The sources of costs and consequences are referenced but only briefly described. No justifications are provided. For FMT, the study uses prices from OpenBiome, Center for Medicare and Medicaid Services Fees Schedule, and the use of average wholesale drug prices. The outcome measure is QALYs, but the utility measures are based on other patient populations than patients with rCDI. This is acknowledged but not further discussed and the utility weights are not included in deterministic sensitivity analyses.
**Question 7.**	**Not applicable.** Costs are reported in 2019 USD. The time horizon of the study is less than a year, why discounting was not indicated. This is noted in the study.
**Question 8.**	**No.** The study applies an incremental analysis of the costs and consequences of two of the alternatives. However, this should supposedly not have been done since FMT by colonoscopy strongly dominates all other treatment options in the base case analysis (Table 3). The result is presented and possibly misinterpreted.
**Question 9.**	**Yes.** The study includes both deterministic (one-way and two-way) and probabilistic sensitivity analyses. The study describes that when ranges are not available, the estimate is varied with what seems to be an arbitrary range of +/- 25 %. No justifications were provided. The study was sensitive to the uncertainties in the parameter estimates for cure rates and costs of FMT by colonoscopy or capsules. In the probabilistic sensitivity analysis, a high probability of FMT being cost-effective was found, but considerable uncertainty was reported between the two delivery methods of FMT.
**Question 10.**	**Cannot tell.** The conclusions of the study are coherent with the results of the study. However, the base case analysis might be misinterpreted regarding the cost-effectiveness of capsules compared with colonoscopy. The results are briefly compared to those of others, but differences are not discussed. The study does not explicitly discuss the generalizability of the study results. Still, it addresses other issues relevant for decision-makers, e.g. differences in strains, contraindications for a colonoscopy, and unknown long-term safety of FMT. Implications of uncertainty for the decision is discussed but could have been elaborated. Suggestions for clinical practice and future research are provided.

**Section/item**	**Abdali et al., 2020 [7]**
**Question 1.**	**Yes.** The study examines both the cost and consequences of several relevant alternatives for the treatment of rCDI. The time horizon is relatively short (1 year). The study applies the perspective of the UK National Health Service (NHS) and includes a description of areas covered by this perspective. The patient population is adequately described but the study could have added a statement on the severity of rCDI. No subgroups are included.
**Question 2.**	**Yes.** The study considers several relevant alternatives for the treatment of rCDI. Different delivery methods for FMT exist (enema and capsules) and could have been included; however, a statement on the omission has been provided. The alternatives are described in sufficient detail to judge the applicability of the analysis. However, the included dosage of vancomycin and the second-line treatment after failure of fidaxomicin is somewhat unclear.
**Question 3.**	**Cannot tell.** The study includes several sources to inform on the effectiveness of the different alternatives. The study includes a pragmatic review and documentation for the search in the supplemental material. This enhances transparency in the identification of the included studies. Also, the study explicitly describes that literature covers patients with rCDI. However, the study does not explain how individual studies were selected for inclusion or how the information from the studies was pooled. References are applied for each input. The included studies are both randomised controlled trials and observational studies. In addition, estimates for recurrence rates for FMT were drawn from other economic evaluations instead of referring to the primary sources.
**Question 4.**	**No.** The study applied a Markov model, which seems reasonable. A 1-year time horizon is used in the analysis. This limits the estimation of the full numbers of QALYs gained from more effective treatments. The study applies a health sector perspective and describes what this entails (direct medical costs, cost of administration and CDI-related hospitalisation). This seems to agree with the included items. However, the study did not cover costs and

	consequences for any adverse events of FMT and did not include outpatient follow-ups. Treatment with FMT consists of the product (including donor selection, testing, preparation and storage), delivery method, and recovery time at the hospital. The pharmacological treatment includes a description of the dosage and treatment regimen. However, it is a bit unclear precisely of what these alternatives consist.
**Question 5.**	**Yes.** The study only partly separates the measurement and valuation costs and consequences.
**Question 6.**	**Yes.** In general, the sources of data are referenced, and brief descriptions of the inputs are provided. The costs of FMT are elaborated; however, the process included in the preparation of FMT material is not described. The costs are drawn from several separate sources including published literature, estimates from local databases and expert estimates. The study uses QALY's as the effectiveness measure, and the utility measures for rCDI are based on EQ-5D-3L estimates drawn from patients with UK inpatients with CDI. The utility value for healthy individuals is drawn from population norms. Utility weights are not included in deterministic sensitivity analyses.
**Question 7.**	**Not applicable.** Costs are reported in 2018 GBP. The time horizon of the study is less than a year, why discounting is not indicated. This is noted in the study.
**Question 8.**	**Yes.** The study includes an incremental analysis of the costs and consequences when indicated.
**Question 9.**	**Yes.** The study includes both deterministic (scenario) and probabilistic sensitivity analyses. The study describes that when ranges are not available, the estimate is varied with what seems to be an arbitrary range of +/- 20 %. The study is not sensitive to changes in specific parameters. In the probabilistic sensitivity analysis, a high probability of FMT being cost-effective is identified, but some uncertainty is found between the two delivery methods of FMT.
**Question 10.**	**Yes.** The study conclusions are based on the study findings. The discussion includes a short description of the results of the primary analysis and probabilistic sensitivity analysis. The study elaborates several strengths and limitations. However, preferably, the study could have been more conservative regarding the effectiveness estimates applied in the study. The study findings are compared to those of others, and some of the discrepancies are highlighted. The study briefly mentions issues of implementation. In addition, a short note on the generalizability and need for future research are provided.

References

1. Konijeti GG, Sauk J, Shrime MG, Gupta M, Ananthakrishnan AN. Cost-effectiveness of competing strategies for management of recurrent clostridium difficile infection: A decision analysis. Clinical Infectious Diseases. 2014;58(11):1507-1514.
2. Varier RU, Biltaji E, Smith KJ, et al. Cost-Effectiveness Analysis of Fecal Microbiota Transplantation for Recurrent Clostridium difficile Infection. Infection Control and Hospital Epidemiology. 2015;36(4):438-444.
3. Lapointe-Shaw L, Tran KL, Coyte PC, et al. Cost-Effectiveness Analysis of Six Strategies to Treat Recurrent Clostridium difficile Infection. PloS one. 2016;11(2):e0149521.
4. Merlo G, Graves N, Brain D, Connelly LB. Economic evaluation of fecal microbiota transplantation for the treatment of recurrent Clostridium difficile infection in Australia. Journal of gastroenterology and hepatology. 2016;31(12):1927-1932.
5. Baro E, Galperine T, Denies F, et al. Cost-Effectiveness Analysis of Five Competing Strategies for the Management of Multiple Recurrent Community-Onset Clostridium difficile Infection in France. PloS one. 2017;12(1):e0170258.
6. Luo Y, Lucas AL, Grinspan AM. Fecal Transplants by Colonoscopy and Capsules Are Cost-Effective Strategies for Treating Recurrent Clostridioides difficile Infection. Digestive diseases and sciences. 2020;65(4):1125-1133.
7. Abdali ZI, Roberts TE, Barton P, Hawkey PM. Economic evaluation of Faecal microbiota transplantation compared to antibiotics for the treatment of recurrent Clostridioides difficile infection. EClinicalMedicine. 2020;24:100420.
8. Drummond MF, Sculpher MJ, Claxton K, Stoddart GL, Torrance GW. *Methods for the Economic Evaluation of Health Care Programmes*. Fourth ed. Oxford University Press; 2015.
