# Supplementary material for: Health economic evaluations comparing faecal microbiota transplantation with antibiotics for treatment of recurrent Clostridioides difficile infection: a systematic review
Source: Health Econ Rev. 2021 Jan 13;11:3. doi: 10.1186/s13561-021-00301-7 (PMC7805077; doi:10.1186/s13561-021-00301-7)
Supplement: Supplementary file 5 — Additional file 5. is a .pdf file which contains a table with the inputs applied in the health economic evaluations. [file 13561_2021_301_MOESM5_ESM.pdf]

Additional file 5: Model inputs

The table includes the parameter inputs reported in the health economic evaluations identified in the systematic review. Total costs for FMT have been calculated by the authors of the systematic review by adding up the separate units reported in the studies. CDI = *Clostridioides difficile* infection rCDI = recurrent *Clostridioides difficile* infection. FMT= faecal microbiota transplantation. AE=adverse events. RR=relative risk.

Input	Konijeti et al., 2014 [1]	Varier et al., 2015 [2]	Lapointe-Shaw et al., 2016 [3]	Merlo et al., 2016 [4]	Baro et al., 2017 [5]	Luo et al., 2019 [6]	Abdali et al., 2020 [7]
**Utility weights**							
Healthy	**0.88** (65y)	**1.00**	**0.91** (70-79y)<br>**0.88** (≥80y)	**0.84** (65y)	**1**	**0.88**	**0.78** (65y)
RCDI	**0.82** (mild or moderate)<br>**0.71** (severe)	**0.88**	**0.70**	**0.88**	**0.782** (mild or moderate)<br>**0.60** (severe)	**0.77**	**0.42** (relapse or recurrence)
Complications	**0.61** (colectomy)<br>**0.86** (post-colectomy)	**0.57** (fulminant colitis)<br>**0.15** (AE of FMT)		**0.536**(colectomy)<br>**0.7** (ileostomy)	**0.536** (post-colectomy)<br>**0.770** (AE of FMT)	**0.536** (post-colectomy)	
**Probabilities for cure (and recurrences)**							
FMT by colonoscopy	**0.945 (0.091)**	**0.910**	**(0.078)**	**3.05** (RR compared with vancomycin)	**0.894** (first FMT)<br>**0.563** (second FMT)<br>**(0.022)**	**0.914 (0.084)**	**0.87** (single)<br>**0.95** (multiple)<br>**(0.09)**
FMT by nasoduodenal/-gastric tube	**0.813 (0.063)**		**(0.233)**	**3.05** (RR compared with vancomycin)	**0.795** (first FMT)<br>**0.750** (second FMT)<br>**(0.021)**		**0.81** (single)<br>**0.88** (multiple)<br>**(0.12)**
FMT by enema	**0.815 (0.091)**		**(0.185)**		**0.833** (first FMT)<br>**0.500** (second FMT)<br>**(0.000)**		
FMT by capsules						**0.830 (0.084)**	
Oral vancomycin	**0.916 (0.355)**		**(0.517)**	**0.308**			**0.31 (0.31)**
Pulse-taper vancomycin	**0.690 (0.274)**	**0.690**	**(0.178)**		**0.771 (0.568)**	**0.776 (0.363)**	**0.58 (0.31)**
Fidaxomicin	**0.937 (0.197)**		**(0.620** RR compared with vancomycin)		**0.812 (0.211)**	**0.885 (0.179)**	**0.42 (0.15)**
Metronidazole	**0.710 (0.421)**		**0.776 (0.40)**				
Bezlotoxumab + vancomycin						**0.800 (0.165)**	
Age dependent RR of recur. per 10y			**(1.16)**				

**Probabilities for death /adverse events**							
Death from CDI or complications	**0.413** (mortality severe CDI, postcolectomy), **0.580** (mortality severe CDI, medical treatment)	**0.0003** (mortality FMT colonoscopy)	**0.002** (70y, mortality all causes), **0.005** (80y, mortality all causes), **0.073** (mortality CDI), **1.41** (RR death CDI per 10y), **0.0006** (mortality FMT colonoscopy) **0.003** (mortality FMT nasogastric tube)	**0.092** (mortality CDI), **0.416** (mortality post-colectomy)	**0.007** (mortality mild CDI), **0.339** (mortality severe uncomplicated CDI), **0.0003** (mortality FMT colonoscopy), **0.0002** (mortality FMT duodenal infusion), **0.407** (mortality post-colectomy)	**0.33** (mortality severe CDI), **0.46** (mortality colectomy)	**0.18** (mortality CDI), **0.002** (65y, mortality other causes).
Complications	**0.180** (severe CDI if failure) **0.280** (colectomy if severe CDI)	**0.16** (fulminant colitis) **0.0028** (AE of FMT)	**0.174** (hospitalisation for CDI)	**0.012**(colectomy) **0.0005** (ileostomy closure) **0.0027** (reinfection) **0.1** (recurrence given reinfection)	**0.318** (colectomy) **0.180** (severe uncomplicated CDI), **0.012** (severe complicated CDI) **0.002** (AE of FMT colonoscopy), **0.0005** (AE of FMT duodenal infusion), **0.00** (hospitalisation mild CDI)	**0.257** (hospitalisation for CDI), **0.192** (colectomy)	
**Costs 2019-level GBP**/original value (resources use included)							
Original cost year	2012	2011	2014	2015	2016	2019	2018
Original currency	US dollars	US dollars	Canadian dollars	Australian dollars	Euro	US dollars	British pound
FMT resource use included:	Vancomycin (500mg qid x 4d), donor and recipient screening, preparation and the procedure	Donor and recipient screening, procedure and facility costs	Vancomycin (125mg qid x 14d), donor and recipient screening, preparation, the procedure, nursing time after the	Vancomycin (125mg x 4-5d), pre-treatment consultation, bowel lavage, donor and recipient screening, donor recruitment,	Vancomycin (500mg qid x 4d), donor screening, two donor outpatient visits, preparation, traceability, bowel	Vancomycin (125mg qid x 10d), ready-to-use microbiota preparation or ready-to-use capsules and, if	Donor and recipient selection and screening, preparation, storage, bowel preparation, the procedure, and

			procedure, three outpatient visits	preparation, storage, the procedure and supervision after the procedure	preparation, the procedure and two follow-up outpatient visits for recipients	applicable, delivery by colonoscopy	recovery time after the procedure
FMT by colonoscopy	**1,704**/2,250 (authors' calculation)	**840**/1,086	**3,158**/5,107 (authors' calculation)	**1,136**/2,251	**1,369**/1,417 (authors' calculation)	**1,816**/2,671	**3,057**/3,006
FMT by nasoduodenal/-gastric tube	**1,629**/2,152 (authors' calculation)		**829**/1,340 (authors' calculation)	**1,106**/2,190	**1,163**/1,204 (authors' calculation)		**753**/740
FMT by enema	**1,554**/2,052 (authors' calculation)		**5,032**/8,137 (authors' calculation)		**1,095**/1,133 (authors' calculation)		
FMT by capsules						**1,326**/1,950	
Oral vancomycin	**515**/680 (125mg qid x 10d) **189**/250 (iv compounded for oral 125mg qid x 10d, if severe CDI)		**215**/347 (125mg qid x 14d)	**332**/658 (125 mg qid x14d) **221**/438 (125 qid x10d, if additional recurrences)	**48**/50 (500mg qid x 10d, if severe complicated CDI)		**203**/200 (250mg qid x 10d) **407**/400 (500mg qid x 10d)
Pulse-taper vancomycin	**644**/850 (125 mg qid x 10d + 125 mg every third day x 10 doses, if additional recurrences)	**1,600**/2,069 (250 mg qid x 14d + 6 weeks taper)	**312**/505 (125 mg qid x 14d + 125 mg bid x 7d + 125mg qd x 7d + 125 mg qod x 7d + 125 mg every third day x 7d, if additional recurrences)		**56**/58 (125mg qid x 10d + 500mg qod x 21d)	**1,729**/2,542 (125 mg qid x 14d + 125 mg bid x 7d + 125 mg qd x 7d + 125 mg qod x 7d + 125 mg every third day x 7d)	**303**/298 (6-weeks taper pulse starting with 250m, if additional recurrences)
Fidaxomicin	**2,120**/2,800 (200mg bid x 10d)		**1,487**/2,405 (200mg bid x 10d)		**1,368**/1,416 (200mg bid x 10d)	**3,155**/4,639 (200mg bid x 10d)	**1,373**/1,350 (200mg x 10d)
Metronidazole	**17**/22 (500 mg tid x 10d) **24**/32 (iv 500mg tid x10d, if severe CDI)		**12**/20 (500mg tid x 6d) **24**/39 (500mg tid x 14d)	**14**/27 (400mg tid x10d, if reinfection)	**11**/11 (iv 500mg tid x10d, if severe complicated CDI)		
Bezlotoxumab						**3,101**/4,560 (bezlotoxumab 10mg/kg one-time infusion and oral vancomycin 125mg qid x 10d)	

Other cost/resource use included	**79**/104 (follow-up patient visit), **11,869**/15,675 (hospitalisation, if severe CDI), **28,492**/37,629 (colectomy), **38**/50 (c. diff. nucleic acid amplification testing)	**18,337**/23,717 (severe, fulminant colitis including hospitalisation, medical therapy and the probability and cost of surgery), **1,651**/2,136 (rCDI including repeat testing and vancomycin taper), **23,248**/30,069 (AE of FMT)	**184**/297 (two outpatient visits for patients treated with medications) **10390**/16800 (hospitalisation CDI)	**2,255**/4,467 (hospitalisation, rCDI3.6 days) **5,856**/11,600 (colectomy) **7,655**/15,165 (ileostomy closure)	**2,116**/2,190 (hospitalisation mild-moderate CDI), **8,126**/8,412 (hospitalisation severe CDI), **695**/719 (colectomy), **273**/283 (AE of FMT colonoscopy), **221**/229 (AE FMT duodenal tube)	**15,178**/22,321 (hospitalisation CDI), **19,345**/28,448 (colectomy)	**2,054**/2,020 (FMT hospitalisation, 5 days), **4,109**/4,040 (antibiotics hospitalisation, 10 days)

References

1. Konijeti GG, Sauk J, Shrime MG, Gupta M, Ananthakrishnan AN. Cost-effectiveness of competing strategies for management of recurrent clostridium difficile infection: A decision analysis. Clinical Infectious Diseases. 2014;58(11):1507-1514.
2. Varier RU, Biltaji E, Smith KJ, et al. Cost-Effectiveness Analysis of Fecal Microbiota Transplantation for Recurrent Clostridium difficile Infection. Infection Control and Hospital Epidemiology. 2015;36(4):438-444.
3. Lapointe-Shaw L, Tran KL, Coyte PC, et al. Cost-Effectiveness Analysis of Six Strategies to Treat Recurrent Clostridium difficile Infection. PloS one. 2016;11(2):e0149521.
4. Merlo G, Graves N, Brain D, Connelly LB. Economic evaluation of fecal microbiota transplantation for the treatment of recurrent Clostridium difficile infection in Australia. Journal of gastroenterology and hepatology. 2016;31(12):1927-1932.
5. Baro E, Galperine T, Denies F, et al. Cost-Effectiveness Analysis of Five Competing Strategies for the Management of Multiple Recurrent Community-Onset Clostridium difficile Infection in France. PloS one. 2017;12(1):e0170258.
6. Luo Y, Lucas AL, Grinspan AM. Fecal Transplants by Colonoscopy and Capsules Are Cost-Effective Strategies for Treating Recurrent Clostridioides difficile Infection. Digestive diseases and sciences. 2020;65(4):1125-1133. doi:10.1007/s10620-019-05821-1
7. Abdali ZI, Roberts TE, Barton P, Hawkey PM. Economic evaluation of Faecal microbiota transplantation compared to antibiotics for the treatment of recurrent Clostridioides difficile infection. EClinicalMedicine. 2020;24:100420.
